# Experimental Research of the Time-Dependent Effects of Steel–Concrete Composite Girder Bridges during Construction and Operation Periods

**DOI:** 10.3390/ma13092123

**Published:** 2020-05-03

**Authors:** Guang-Ming Wang, Li Zhu, Guang-Pan Zhou, Bing Han, Wen-Yu Ji

**Affiliations:** 1 School of Civil Engineering, Beijing Jiaotong University, Beijing 100044, China; 14115279@bjtu.edu.cn (G.-M.W.); skyiflv@163.com (B.H.); wyji@bjtu.edu.cn (W.-Y.J.); 2 School of Science, Nanjing University of Science and Technology, Nanjing 210094, China; newsypaper@163.com

**Keywords:** steel–concrete composite bridge, I-shaped beam, concrete creep, temperature, prediction, experiment

## Abstract

The present work aimed to study the effects of temperature changes and concrete creep on I-shaped steel–concrete composite continuous girder bridges during construction and operation processes. This study combined structural health monitoring data, an ANSYS finite element simulation, and the age-adjusted effective modulus method to obtain the variation laws of temperature and internal force in composite girders. Moreover, a temperature gradient model was proposed that is suitable for bridges in Hebei, China. In addition, a concrete creep experiment under unidirectional axial compression was performed using concrete specimens prepared from the concrete batch used to create the composite girder. The long-term evolution laws of the deflection and internal force of the composite girder were obtained by predicting the concrete creep effect. The measured data showed that the temperature variation trends of the steel beam and concrete slab were characterized by a sinusoidal curve without a temperature lag. The heating rate of the concrete slab was higher than the cooling rate. The prediction results showed that the internal force changes in the composite girder were characterized by three stages. The stress changes in the composite girder during the first 10 days were significant and the stress charge rate of the concrete slab, the steel girder and the shear stud can reach 5%–28%. The stress change rate decreased continuously during 10–90 days. The stress changed slowly and smoothly after 90 days. This research can provide feedback and reference for structural health monitoring and service safety control of similar I-shaped steel–concrete composite bridges.

## 1. Introduction

Steel–concrete composite girder bridges are becoming increasingly popular with increasing bridge spans and advantages in terms of reliable performance, reasonable cost, and structural rationality. However, some shortcomings of these structures need to be solved, including the stress redistribution caused by ambient temperature changes and concrete shrinkage and creep. Therefore, the time-dependent effects and the evolution law of structural performance during bridge construction and operation periods should be studied, which is beneficial to the further promotion and application of composite girder bridges [1].

First, the maximum stress caused by temperature changes can reach 20%–30% of the allowable stress, which cannot be ignored [2]. Moreover, thermal residual stresses and relative slip between steel and concrete will be caused by the temperature difference between the steel beam and concrete slab [3,4,5]. Chen [6], Gu [7], Zhou [8], and Liu [9] deduced formulas for the internal force and relative slip in steel–concrete composite girders under the influence of temperature. Chen [10], Su [11], Wang [12], and Xiao [13] verified the existence of a large temperature difference and gradient in a steel–concrete composite box girder based on monitoring data of the temperature field, and the temperature distribution was different from that in the existing Chinese specifications. Moreover, the daily temperature differences, convection, and solar radiation were the main factors affecting the temperature field of the composite girder. However, the above studies were mainly directed to the single-piece I-beam girders or steel box girders, whereas few studies involved multiple I-beam girders. Furthermore, only the calculation method for the temperature difference between the steel and concrete is regulated by the Chinese regulations. The influences of the variation of the transverse temperature on the mechanical behavior of the bridges remain to be further studied.

The theories describing the stress–strain constitutive relationship caused by concrete creep include the aging theory (creep rate method), elastic creep theory (superposition method), elastic aging theory (flow rate method), secondary effect flow theory, and age adjustment effective modulus method [14,15,16,17,18]. The concrete creep effects of composite bridges were analyzed by several researchers considering the shear connections, the shear lag effect in box girders, the randomness of shrinkage and creep effects, and the changes in temperature and humidity [19,20,21,22,23,24,25,26]. In terms of experimental research, Gara, Ranzi, and Sullivan obtained an analysis model of concrete creep by conducting long-term full-scale tests with simply supported composite girder bridges [27,28,29,30]. Fan and Nie studied the concrete shrinkage and creep effects and cracking of composite girder bridges based on long-term loading tests and proposed an improved composite deck system for cable-stayed bridges [31,32]. However, the above studies were mainly aimed at analyzing the concrete shrinkage and creep effects of composite girders under specific conditions. The establishment of concrete constitutive relations and the realization of corresponding numerical models still needs to be analyzed. Numerical simulation and scale model test methods are mainly used in research. However, there are few studies aimed at long-term monitoring of concrete shrinkage and creep effects for real bridges because of the difficulty in long-term field monitoring.

In addition, the significances of the long-term monitoring of bridges are as follows: (1) the difference between the monitored experimental results and the numerical model for prediction analysis of bridges can be identified to correct the model. The corrected model can be further applied in the subsequent analysis of the mechanical behavior of the bridges. (2) The bridges are monitored for the long term to ensure their safety. This can provide a steady foundation for superior behavior of bridges in their life cycle and meet the requirement of the long-cycle design idea. (3) The monitored experimental results of the mechanical behavior of the bridges in a complicated and variable environment may create a new research issue. Therefore, long-term monitoring of the bridges is important for the development of investigation of the bridges.

In this paper, the effects of temperature changes and concrete creep on I-shaped steel–concrete composite continuous girder bridges were studied. This study combined field monitoring data, an ANSYS finite element (FE) simulation, and the age-adjusted effective modulus method to obtain the variation laws of temperature and internal force of the composite girders. Moreover, a temperature gradient model suitable for bridges in the Hebei region of China was proposed. In addition, a concrete creep experiment under unidirectional axial compression was carried out using concrete material from the batch used to create the composite girder. Then, concrete creep curves under 28-day loading were obtained. The long-term evolution laws of the deflection and internal force of the composite girder in response to concrete creep were predicted.

## 2. Background Project Overview

### 2.1. Bridge Overview

**Figure 1:**
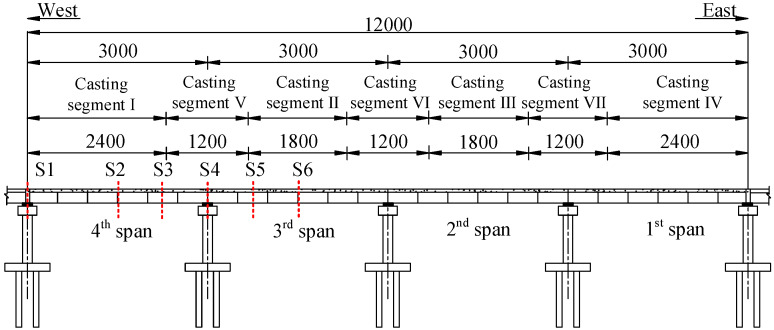
Layout of the first west standard segment of the bridge (units: cm).

The background project is located in Baoding city, Hebei Province in China, and the first west segment of the bridge is shown in Figure 1. Sections S1, S2, S3, S4, S5, and S6 in Figure 1 represent the control sections located at the side support, 1/2 and 3/4 sections of the 4th span, the 2nd pier top, 1/4 and 1/2 sections of the 3rd span, respectively.

**Figure 2:**
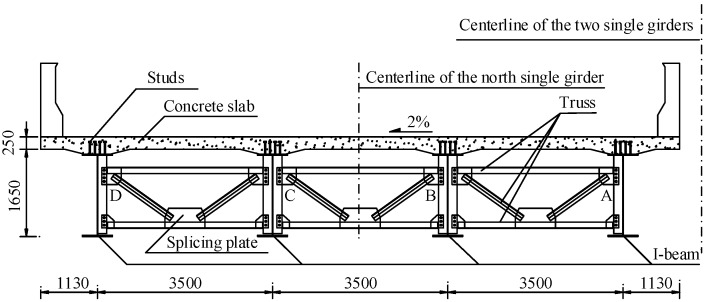
Cross-section of the north half composite girder (units: mm).

The I-shaped steel–concrete composite continuous girder was used in the superstructure of the bridge. The north and south side girders are symmetrical relative to the road centerline. The cross-section of the northern half of the composite girder is shown in Figure 2. The steel beams are labeled A, B, C, and D from the road centerline to the outside. The thickness of the web of the steel beam was 12 mm.

### 2.2. Structural Health Monitoring (SHM) System

**Figure 3:**
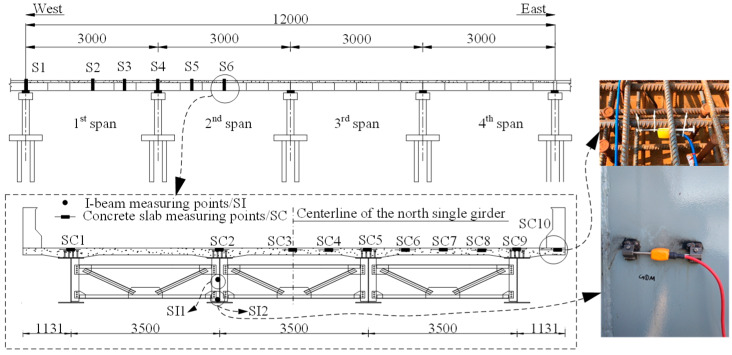
Layout of the strain gauges for the steel beam web and concrete slab (units: cm).

**Figure 4:**
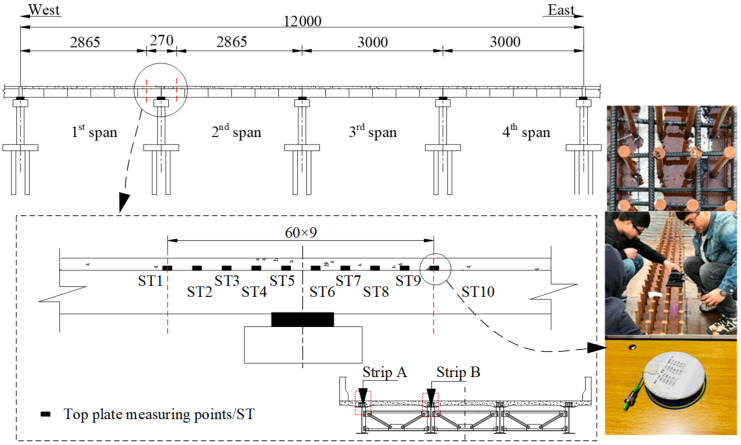
Layout of the fiber Bragg grating sensors for the top plate of the steel beam (units: cm).

**Figure 5:**
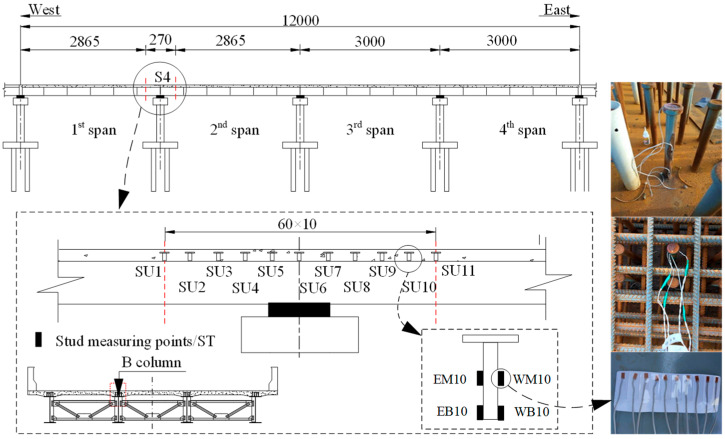
Layout of the strain gauges for the studs (units: cm).

**Figure 6:**
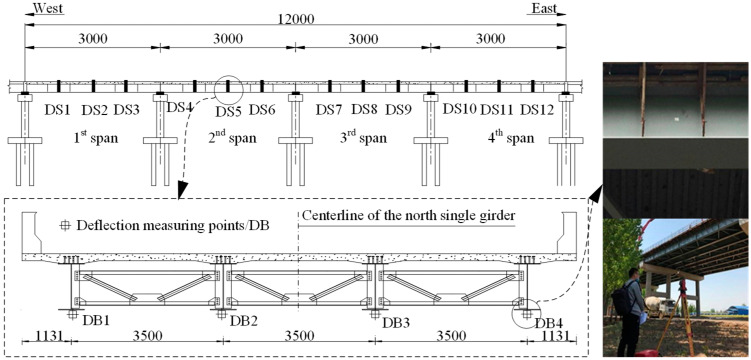
Layout of the measuring points for girder deflection (units: cm).

Figure 3 shows the arrangement of the vibrating string strain gauges attached to the steel beam web and the ones embedded in the concrete slab in Sections S1–S6. The arrangement of the fiber Bragg grating sensors attached to the top flange surface of the steel beam is shown in Figure 4. In addition, 11 studs symmetrical to S4 were selected, as shown in Figure 5. For the girder deflection, four measuring points were uniformly arranged in the transverse direction for each span, as shown in Figure 6.

## 3. Thermal Effect during the Construction Process

The regulations of the temperature gradient model in the codes of every country are different because the selection of the temperature gradient model is related to the bridge type, cross-section form, sunshine conditions, and geographical location. In this paper, the temperature distribution law of a multiple I-steel composite girder was obtained based on field-measured data, and the most unfavorable temperature gradient mode was determined. Moreover, the transverse gradient model of temperature was proposed, which can guide structural designs.

### 3.1. Variation Law of Girder Temperature

**Figure 7:**
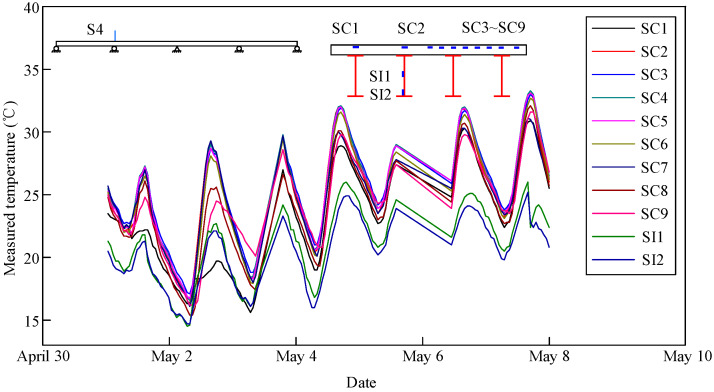
Measured temperature changes in Section S4.

**Figure 8:**
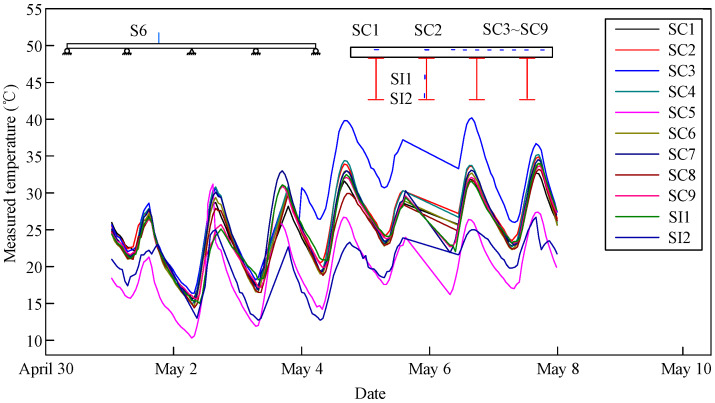
Measured temperature changes in Section S6.

The measured temperature changes in the composite girder in Sections S4 and S6 from May 1 to May 8 of 2018 are shown in Figure 7 and Figure 8, respectively. The temperature variation at each measuring point was characterized by an obvious sinusoidal performance with peak and valley values occurring on each day. Moreover, the temperature change trends were consistent without a lag phenomenon. In addition, the heating rate of the concrete slab was greater than the cooling rate. The fastest heating and cooling rates were 1.5 °C/h at 13:00 and −0.9 °C/h at 21:00, respectively, and the trends of both increased first and then decreased. Moreover, there was a gradient change along the vertical direction of the composite girder. The temperature of the concrete slab was higher than the temperature of the steel beam. The temperature at the middle of the steel beam was higher than the temperature at the bottom flange of the steel beam.

### 3.2. Variation Law of Girder Strain

**Figure 9:**
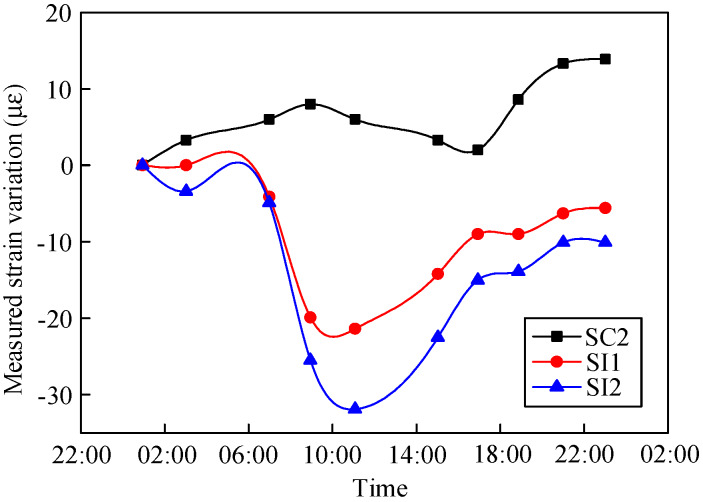
Measured girder strain in Section S4.

**Figure 10:**
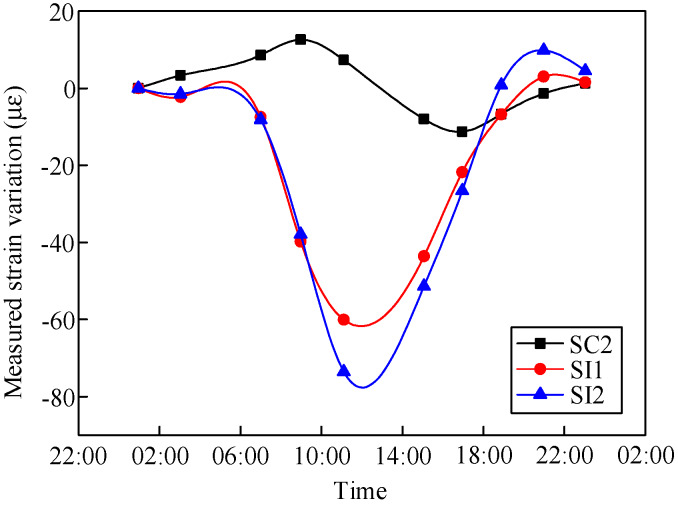
Measured girder strain in Section S6.

The measured strain changes in the composite girder in Sections S4 and S6 in one day are shown in Figure 9 and Figure 10, respectively. Figure 9 shows that the most prominent daily strain variation in the middle of the steel beam was 30.9 με. The daily strain variation in the concrete slabs was smaller: 8 με. Figure 10 shows that the most prominent daily strain variation in the middle of the steel beam was 83.4 με, and that of the concrete slabs was 23.9 με. The daily strain change in the composite girder in Section S4 was smaller than that in Section S6. In addition, the changes in ambient temperature could not be reflected by the embedded vibrating wire sensor in the concrete, and the strain changes in the concrete had a lag of 4.5 h with respect to one of the steel beams.

**Figure 11:**
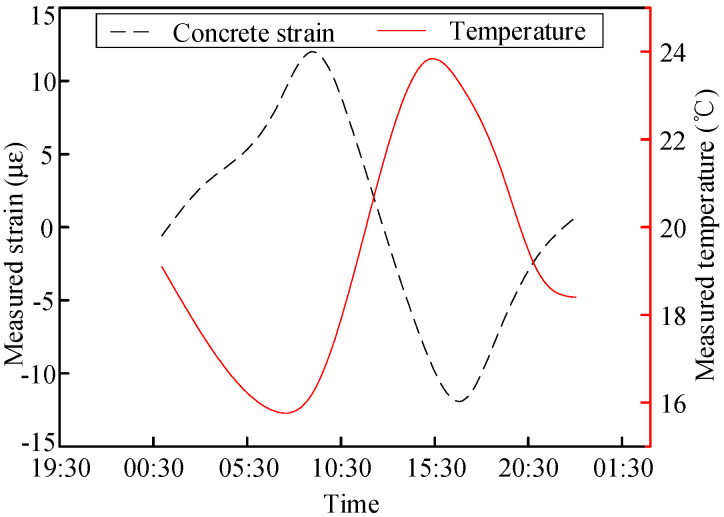
Measured variation in the concrete strain in Section S6 along with temperature changes.

**Figure 12:**
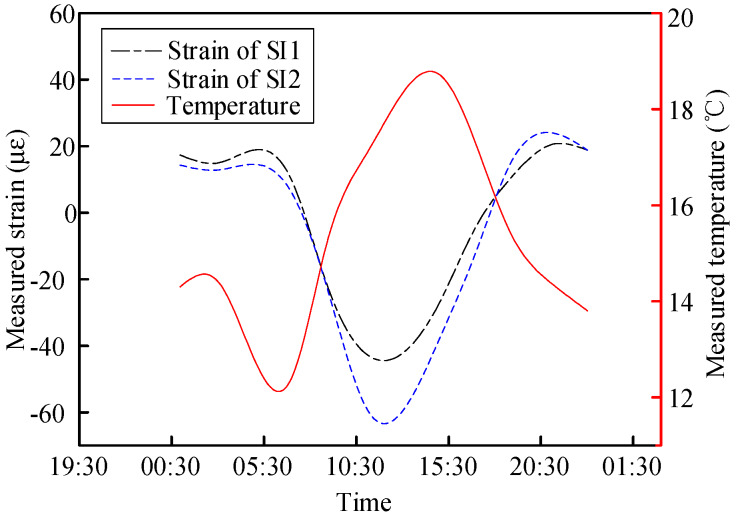
Measured variation in the steel beam strain in Section S6 along with temperature changes.

The variation laws of the internal forces of the concrete slab and steel beam in Section S6 along with temperature changes in one day were analyzed, as shown in Figure 11 and Figure 12, respectively. The compressive strains in the concrete and steel beams were caused by the expansion of material with increasing temperature. The tensile strains were caused by material shrinkage along with decreasing temperature. The strain variation rates in the concrete and steel beams were 3.4 με/°C and 23.9 με/°C, respectively.

**Figure 13:**
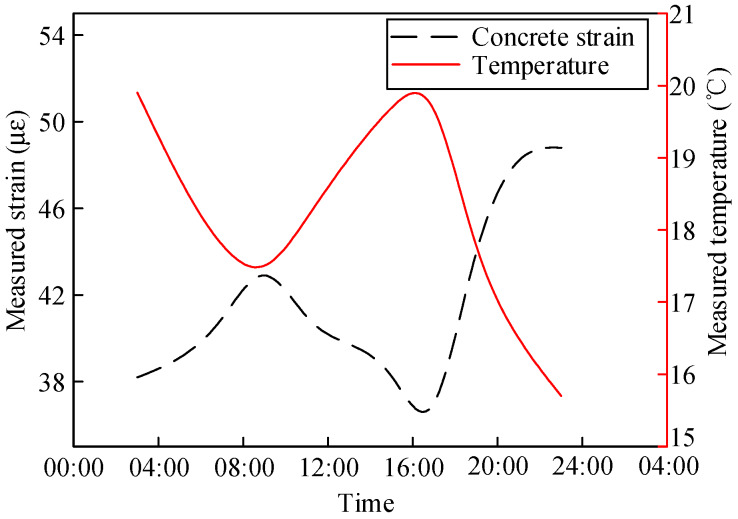
Measured variation in the concrete strain in Section S4 along with temperature changes.

**Figure 14:**
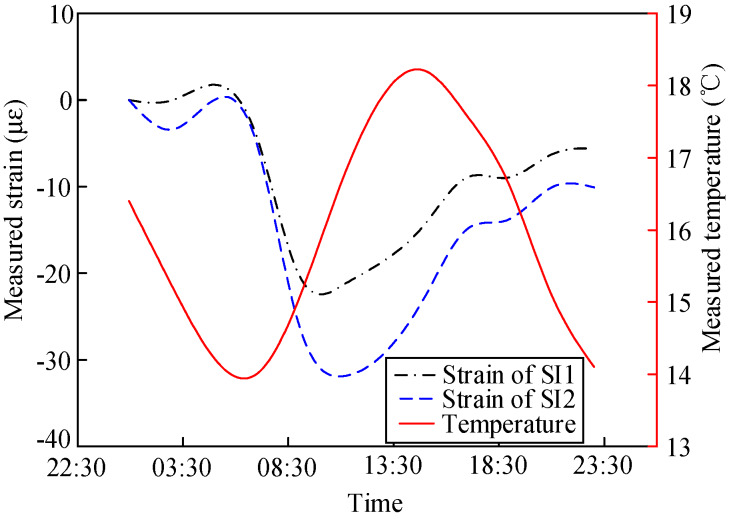
Measured variation in the steel beam strain in Section S4 along with temperature changes.

The variation laws of the internal forces of the concrete slab and steel beam in Section S4 along with temperature changes in one day are shown in Figure 13 and Figure 14, respectively. The strain variation rates in the concrete and steel beams were 5 με/°C and 23.8 με/°C, respectively. 

### 3.3. Proposed Gradient Model of Temperature

The temperature of the concrete slab is characterized by a large transverse gradient because of the bridge direction and environmental shielding. However, the Chinese code specifies only the transverse temperature effect of the wide box girder without cantilevers. Therefore, the suggested temperature gradient model for composite girder bridges in the Hebei Province of China was proposed by processing the temperature monitoring data with a guaranteed rate of 95%.

**Figure 15:**
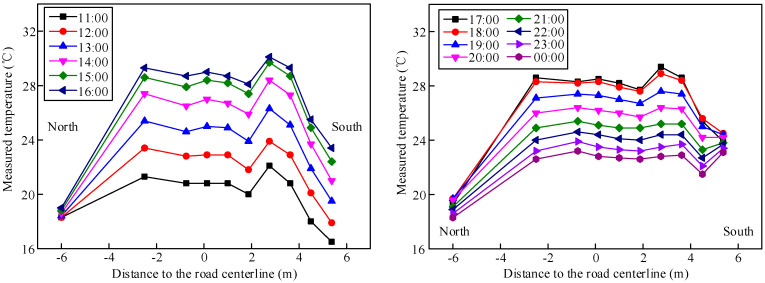
Transverse distribution of concrete temperature in Section S4 for one day.

The transverse distribution of the concrete temperature in Section S4 is shown in Figure 15. The temperature difference along the transverse direction under normal climatic conditions between 1:00 and 11:00 was less than 1.5 °C. The transverse distribution of the concrete temperature from 11:00 to 16:00 was characterized by an isosceles trapezoidal distribution. Moreover, the temperature in the middle region of the concrete slab was higher than that in the outside region of the concrete slab. The transverse distribution of temperature in the south concrete slab gradually became a trapezoidal distribution with increasing temperature due to the long exposure to sunshine. The transverse difference in temperature reached a maximum of 10.1 °C at 17:00. The north measuring points of the concrete were shielded by the steel beams and could not be illuminated by the sun. Therefore, the temperature at these points was the lowest among the measuring points, and the temperatures at these points changed slightly.

The maximum differences in the girder temperatures during each day were extracted as shown in Table 1, and the mean value at each measured point was calculated.

The temperature gradient model for the composite girder bridges in Hebei, China, was proposed with a guaranteed rate of 95%. A temperature gradient of 4 °C/m was adopted for the height range from the bottom of the steel beam to the junction of the flange and web. A temperature gradient of 20 °C/m was adopted for the height range from the junction of the flange and web to the top of the concrete slab. Moreover, the temperature gradient in the pavement could be specified according to the Chinese code. In addition, the heat diffusion and conduction in the girder changed along the transverse direction because of the different boundary conditions, which resulted in the transverse gradient of the temperature. A transverse gradient of 3 °C/m could be adopted. The proposed temperature gradient model can be referenced during the design process.

## 4. Concrete Creep Effect during the Construction and Operation Periods

The stress redistribution in composite girders is difficult to solve by theoretical analysis alone because of the complexity of concrete creep and the property differences between steel and concrete. The variation laws of girder deflection and stress caused by concrete creep were studied based on field monitoring data, concrete creep experiments, and numerical models. 

The expander was added into the concrete of the composite bridge to eliminate the shrinkage effect. Little concrete shrinkage strain was observed in the shrinkage tests of concrete batches. Therefore, only the concrete creep effect was taken into account in the below-mentioned discussion.

### 4.1. Concrete Creep Experiment

#### 4.1.1. Concrete Specimen

A concrete creep experiment under unidirectional axial compression, including two ordinary concrete specimens and two steel fiber reinforced concrete specimens, was carried out using specimens made from the same batch of materials as the actual bridge. The dimension of the concrete specimens was 100 mm × 100 mm × 300 mm. The average value was taken from the 28-day creep data of the two concrete specimens. Moreover, two compensation specimens were designed to offset the deformations caused by the hydration of cement, external temperature, and humidity, including concrete condensation, hardening shrinkage, drying shrinkage, and temperature expansion.

The load on the composite girder during the normal use stage was approximately 40% of the ultimate load, which was selected as the load in the test. The 28-day compressive strength of the ordinary concrete specimen was 55.60 MPa and the 28-day compressive strength of the steel fiber reinforced concrete was 48.40 MPa.

#### 4.1.2. Creep Experiment Process

**Figure 16:**
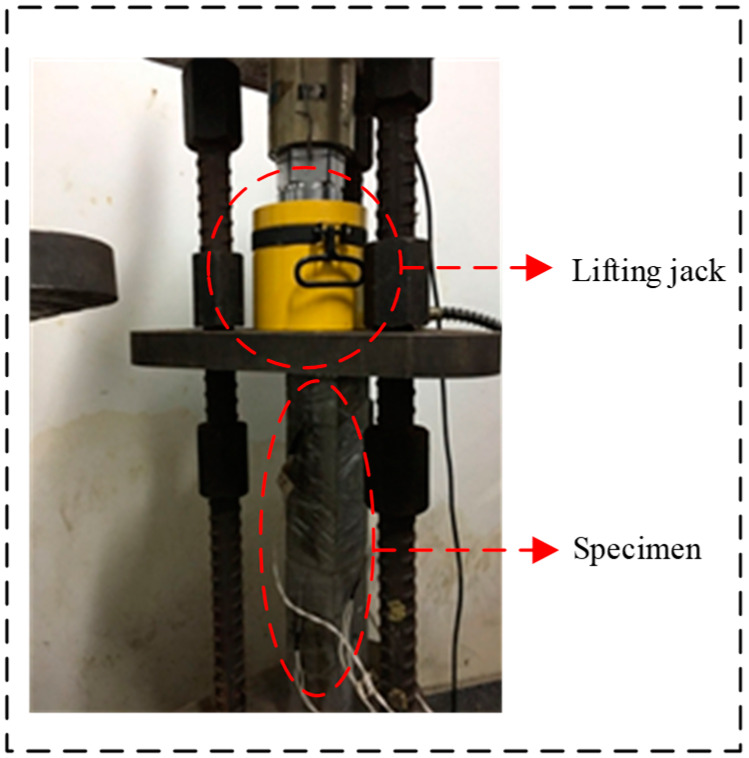
Photograph of a concrete creep experiment.

A picture of the concrete creep experiment is shown in Figure 16. The experimental equipment included a creeper, an oil pressure jack, a pressure sensor, and a static pressure collector. The maximum pressure of the jack was 1000 kN. The sensor used as the load measuring device had a range of 700 kN and an accuracy of 0.01 kN. A paper-based strain gauge with a range of 10 cm and an accuracy of ±1 με was used, and the range was greater than the ultimate strain of the concrete. In addition, the load loss and stress decrease would be caused by concrete shrinkage and creep. Therefore, the accuracy of the experimental data was guaranteed by checking the load regularly, and the jack was used to adjust the load to ensure that the load change was always within ±2%. The loading age of all concrete specimens *t*_0_ was 28 days.

#### 4.1.3. Experimental Results

According to the definition of creep degree function *C*(*t*,*t*_0_), as shown in Equation (1):

(1) $$C\left(t,t_{0}\right)=\frac{\varepsilon_{c}\left(t\right)}{\sigma \left(t_{0}\right)}$$

where *ε*_c_(*t*) is concrete creep strain at time *t*, *σ*(*t*) is concrete stress at time *t*.

**Figure 17:**
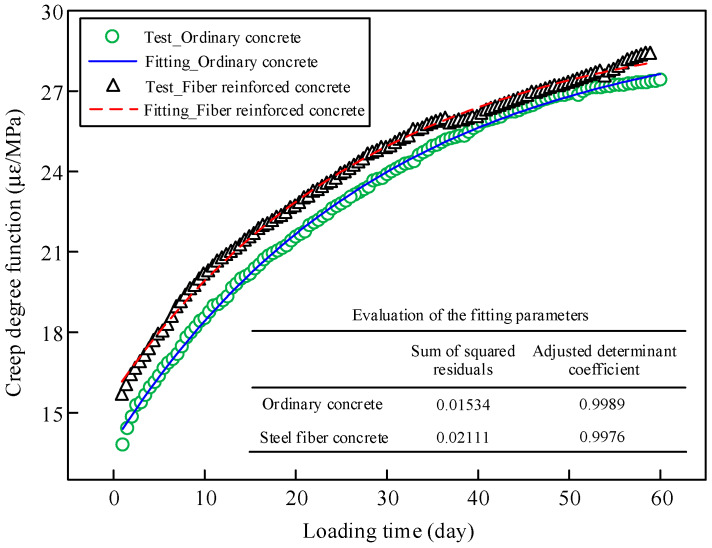
Comparison between the fitting results and the experimental results of the creep degree function.

The curves of the creep degree function *C* of the ordinary concrete and the fiber-reinforced concrete were obtained and shown in Figure 17.

A parameter regression was performed by utilizing a large number of experimental data with high prediction accuracy. A simplified fitting formula of creep degree function was proposed based on the measured data:

(2) $$C\left(t,t_{0}\right)=A_{0}+A_{1}e^{-\left(t-t_{0}\right)/m}$$

where *A*_0_, *A*_1_, and *m* are the parameters that can be regressed by experimental results, which are shown in Table 2. *t*_0_ is the initial loading age of concrete, which is 28 days for the concrete of the bridge.

A comparison between the fitting results of the simplified formula and the experimental data for the creep degree function is shown in Figure 17. The statistical results of the fitting parameters are also given in the figure. The dispersion of the parameters was small. The fitting results are credible.

### 4.2. Prediction Method of the Concrete Creep Effect

The aging coefficient was used to consider the effect of concrete aging on the final creep value based on the age-adjusted effective modulus method [33,34,35,36]. The creep effect of concrete can be accurately calculated by combining the age-adjusted effective modulus method and the FE method. The age-adjusted effective modulus method is expressed as follows:

The sum of elastic strain and creep strain of concrete *ε*(*t*,*t*_0_) is as follows:

(3) $$\begin{array}{cc} \varepsilon \left(t,t_{0}\right) & =\frac{\sigma \left(t\right)}{E_{t_{0}}}+\frac{\sigma \left(t_{0}\right)}{E_{t_{0}}}\varphi \left(t,t_{0}\right)+\frac{1}{E_{t_{0}}}\int_{t_{0}}^{t}\frac{d\sigma \left(\tau \right)}{d\tau }\varphi \left(t,\tau \right)d\tau \\ & =\frac{\sigma \left(t\right)}{E_{t_{0}}}+\frac{\sigma \left(t_{0}\right)}{E_{t_{0}}}\varphi \left(t,t_{0}\right)+\frac{1}{E_{t_{0}}}\int_{\sigma \left(t_{0}\right)}^{\sigma \left(t\right)}\varphi \left(t,\tau \right)d\sigma \left(\tau \right) \end{array}$$

where $E_{t_{0}}$ is Young’s Modulus of concrete at loading time *t*_0_; *φ*(*t*,*t*_0_) is creep coefficient at time *t* with loading time *t*_0_, which can be calculated by Equation (4):

(4) $$\varphi \left(t,t_{0}\right)=C\left(t,t_{0}\right)E_{t_{0}}$$

According to the integral mean value theorem, Equation (3) can be converted into the following form:

(5) $$\begin{array}{cc} \varepsilon \left(t,t_{0}\right) & =\frac{\sigma \left(t\right)}{E_{t_{0}}}+\frac{\sigma \left(t_{0}\right)}{E_{t_{0}}}\varphi \left(t,t_{0}\right)+\frac{\sigma \left(t\right)}{E_{t_{0}}}\varphi \left(t,t_{0}\right)\chi \left(t,t_{0}\right)\\ & =\frac{\sigma \left(t_{0}\right)}{E_{t_{0}}}\varphi \left(t,t_{0}\right)+\frac{\sigma \left(t\right)}{E_{t_{0}}}\left(1+\varphi \left(t,t_{0}\right)\chi \left(t,t_{0}\right)\right)\\ & =\frac{\sigma \left(t_{0}\right)}{E_{t_{0}}}\varphi \left(t,t_{0}\right)+\frac{\sigma \left(t\right)}{E_{\varphi }} \end{array}$$

where *E_φ_* is the age-adjusted effective modulus. The age-adjusted effective modulus *E_φ_* can be expressed as follows:

(6) $$E_{\varphi }=\frac{E_{t_{0}}}{1+\varphi \left(t,t_{0}\right)\chi \left(t,t_{0}\right)}$$

where *χ*(*t*, *t*_0_) is the aging coefficient, which ranges from 0.5 to 1.0 and is generally chosen as 0.8.

According to the proposed creep degree function and the Dischinger method, the aging coefficient can be expressed as follows:

(7) $$\chi \left(t,t_{0}\right)=\frac{1}{1-e^{-\varphi \left(t,t_{0}\right)}}-\frac{1}{\varphi \left(t,t_{0}\right)}$$

### 4.3. Elaborate FE Model

**Figure 18:**
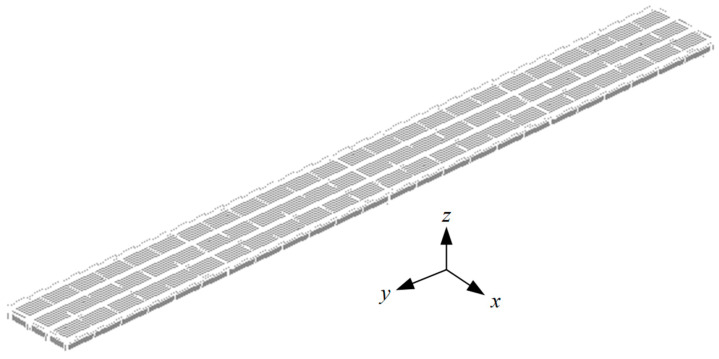
Elaborate FE model of the bridge.

The elaborate FE model of the bridge established in ANSYS is shown in Figure 18. The basic assumptions during model establishment were as follows: the concrete slabs were connected with the steel beams through the studs and only the self-weight of the newly poured concrete slab was considered (i.e., the stiffness was not considered). SHELL181 elements were used to simulate the steel beam and concrete slab. BEAM188 elements were used to simulate the stud and steel truss. The full bridge model included 24,764 nodes and 27,750 elements.

The Young’s modulus of steel is 2.06 × 10^5^ MPa, the Poisson’s ratio of steel is 0.3, and the density of steel is 7850 kg/m^3^. The Poisson’s ratio of concrete is 0.2, and the density of concrete is 2500 kg/m^3^. The age-adjusted effective modulus method was used to simulate the concrete creep effect of the composite girder bridge. The 28-day Young’s modulus of the concrete (3.65 × 10^4^ MPa) was measured by testing specimens made from the same batch of concrete material.

The FE model was verified based on the monitoring data of structural deformation and stress during the concrete pouring process. The pouring dates of each segment are shown in Table 3 and the positions of each segment are shown in Figure 1.

The concrete construction process was simulated based on the FE model by locking and activating the concrete slab elements of each pouring segment in sequence using the life-and-death element method.

**Figure 19:**
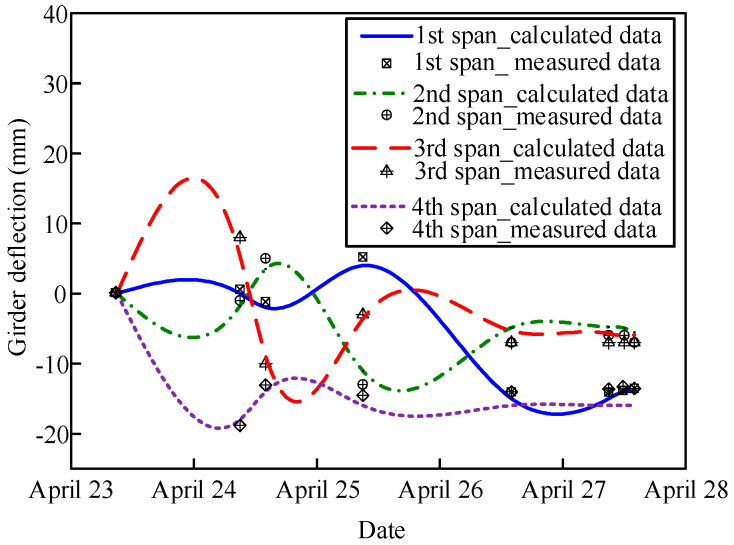
Comparison between the calculated and measured deflections at the middle of each span.

A comparison between the calculated and measured deflections of the steel beams at the middle of each span is shown in Figure 19. The calculated deflections were in good agreement with the measured data with an error of 15%–21%. Moreover, the calculated deflections were slightly smaller than the measured data because that part of the stiffness of the concrete slab was taken into account in the FE model.

**Figure 20:**
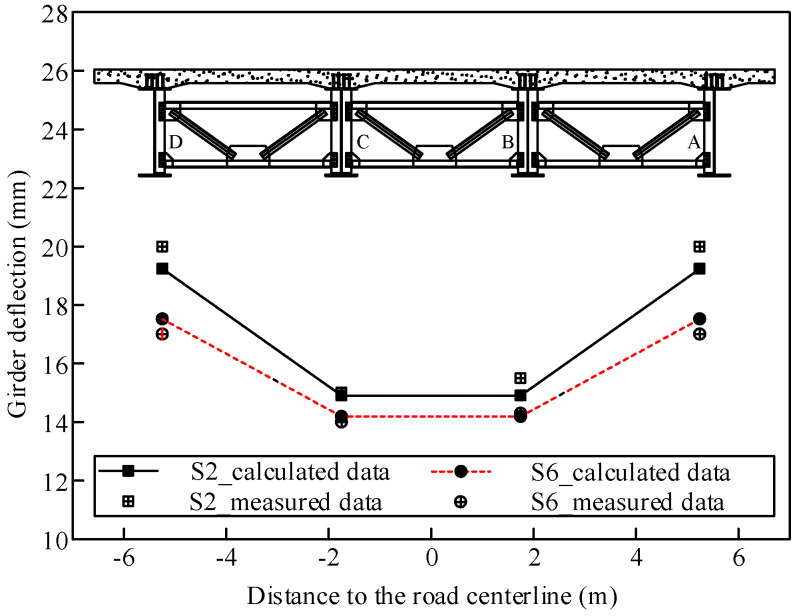
Comparison between the calculated and measured deflection variations along the transverse direction.

Figure 20 shows a comparison of the deflection variation along the transverse direction in Sections S2 and S6 when pouring the concrete in the middle positive moment zone. Due to the structural symmetry, the deflection changes in the inner two steel beams (B and C) were the same, and those of the outer two steel beams (A and D) were the same. Moreover, the deflection changes in the inner and outer beams were different with a difference of 3–4 mm because of the different constraints of the steel beams. 

Comparisons between the calculated and measured girder strains in Sections S4 and S6 are shown in Table 4 and Table 5, respectively. The deviation between the calculated and measured strains was small during the construction stage characterized by large deformation. However, the strain errors during the construction stage characterized by smaller deformation were relatively large because of environmental interference.

**Figure 21:**
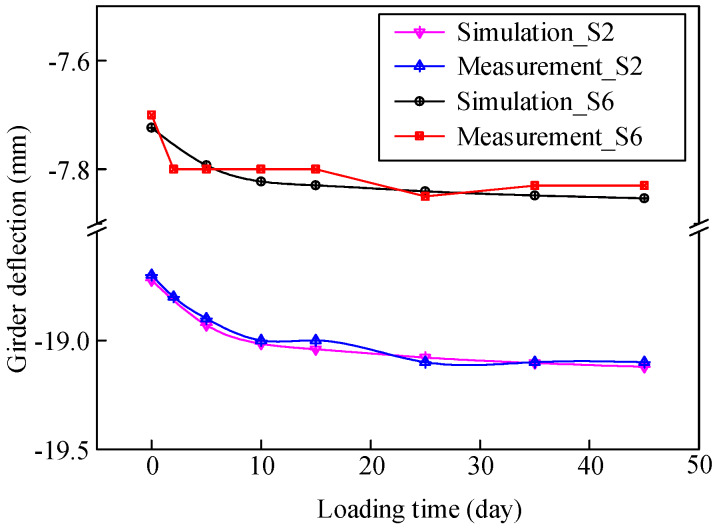
Comparison between the calculated and measured girder deflections within two months after bridge completion.

The deflection monitoring data within two months after bridge completion were used to verify the FE model. Comparisons between the calculated and measured deflections in Sections S2 and S6 are shown in Figure 21. The calculated variation trend of deflection was basically consistent with that of the monitoring results, which reflects the accuracy of the numerical simulation method. In addition, the simulation and monitoring results show that the girder deflection increased continuously in response to the concrete creep effect. Moreover, the deflection variation in Section S2 was larger than that in Section S6.

### 4.4. Predicted Long-Term Effects of Concrete Creep

**Figure 22:**
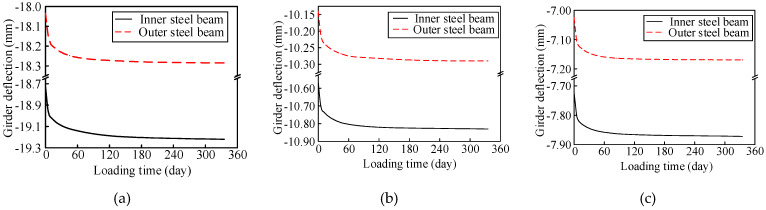
Girder deflection in each section caused by concrete creep (**a**) Section S2; (**b**) Section S3; (**c**) Section S6.

The abovementioned FE model was used to predict the long-term evolution of the structural performance of the composite girder bridge. The prediction results of girder deflection under self-weight are shown in Figure 22, which were characterized by two development stages. The change in girder deflection was fast during the first 15 days and then slow afterwards. Moreover, the change trends and amplitudes of the deflection of the inner and outer steel beams were basically the same. In addition, the deflection change in the second span under the influence of concrete creep was smaller than that in the first span affected by the self-weight of the adjacent span.

**Figure 23:**
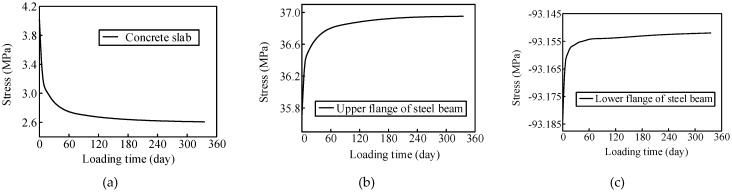
Girder stress changes in Section S4 (**a**) concrete slab; (**b**) upper flange of the steel beam; (**c**) lower flange of the steel beam.

**Figure 24:**
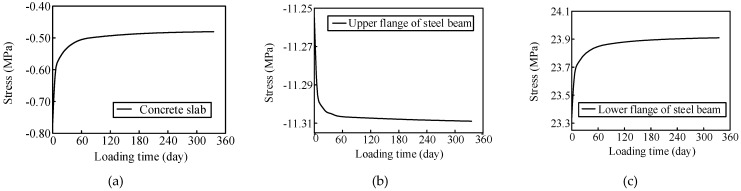
Girder stress changes in Section S6 (**a**) concrete slab; (**b**) upper flange of the steel beam; (**c**) lower flange of the steel beam.

The concrete creep significantly affected the stress redistribution between the steel beam and concrete slab. The girder stress changes in Sections S4 and S6 were predicted as shown in Figure 23 and Figure 24, respectively. The compressive stress in the concrete slab in Section S4 decreased over time. The tensile stress in the upper flange of the steel beam and the compressive stress in the lower flange of the steel beam increased. The compressive stress in the concrete slab in Section S6 increased gradually over time. The compressive stress in the upper flange of the steel beam decreased, and the tensile stress in the lower flange of the steel beam increased.

The stress in the concrete slab in the negative moment zone of the composite girder decreased under the influence of concrete creep. Over time, the steel beams participated more in bearing the load. The concrete slab in the positive bending moment zone was continuously involved in load-bearing, and the compression part of the steel beam decreased gradually. The internal force in the lower flange of the steel beam in the positive moment region still increased due to the stiffness reduction in the negative moment region. In addition, the stress variation in the composite girder was characterized by three stages. The stress variations during the first 10 days were the most significant, the stress change rate decreased continuously during days 10–90, and the stress changed slowly and smoothly after 90 days.

**Figure 25:**
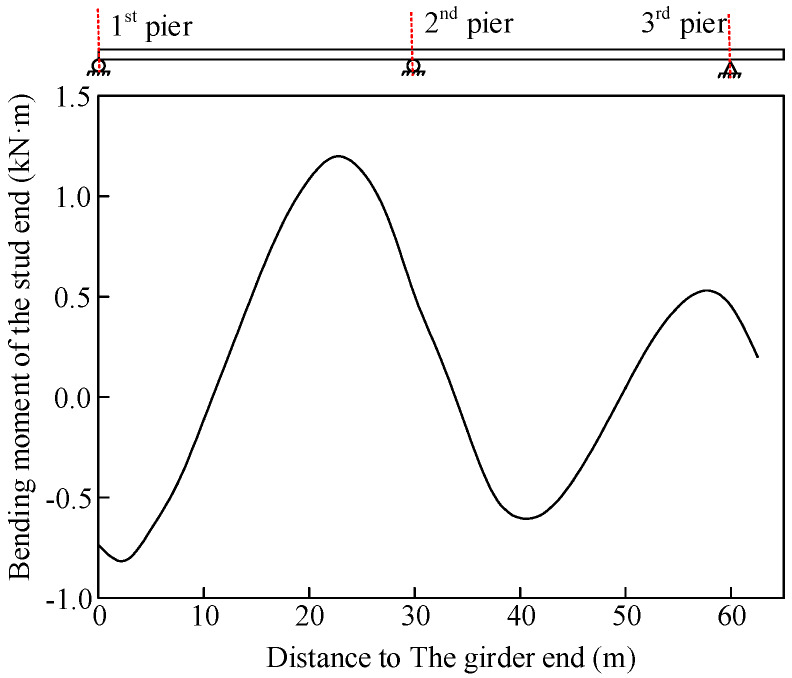
Bending moment of the stud end under self-weight.

**Figure 26:**
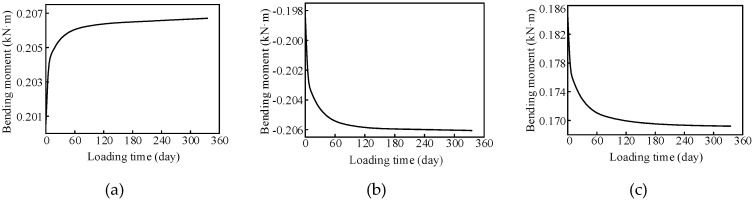
Long-term changes in the stud stress in each section (**a**) stud in the 3rd pier section; (**b**) stud in Section S6; (**c**) stud in Section S4.

Beam elements were used to simulate the stud to consider the interfacial slip. The internal force changes in the studs of the same column at the first and second spans were predicted under concrete creep. The bending moment of the lower end of the stud after 28 days of concrete pouring under self-weight is shown in Figure 25. The bending moment change in the stud along the longitudinal direction was characterized by a sinusoidal distribution. Moreover, the stud bending moment was the largest in Section S3. The long-term variation in the stud bending moment at each section is shown in Figure 26. The stud bending moment near the 3rd pier section increased gradually as a result of concrete creep, whereas the stud bending moments in the other sections decreased gradually.

## 5. Conclusions

In this study, we focused on the influence of time-dependent effects on an I-shaped steel–concrete composite continuous girder bridge, including temperature changes and concrete creep effect. The field monitoring data and FE simulation were combined. The main contributions of this paper are as follows:

(1) The daily temperature variations in the composite girder and the corresponding influence law were studied. The temperature variation trends of the steel beam and concrete slab were characterized by sinusoidal curves without temperature lag. In addition, the heating rate of the concrete slab was higher than the cooling rate. The rates of heating and cooling of the concrete slab first increased and then decreased. Moreover, the suggested temperature gradient model for composite girder bridges in the Hebei Province of China was proposed with a guaranteed rate of 95% by processing the maximum temperature difference during a single day.
(2) A concrete creep experiment under uniaxial compression was carried out using the same batch of concrete material as that used for the bridge. A creep degree function of concrete was obtained. The concrete creep effect of the composite girder bridge was predicted by integrating the age-adjusted effective modulus method and FE model. The accuracy of the numerical analysis was verified by comparing the calculated and monitoring results of girder deflections and strains. The prediction results show that the internal force of the concrete slab in the negative bending moment zone decreased, and the stress of the steel beam increased gradually. The concrete slab in the positive bending moment zone was continuously involved in load bearing. The compression part of the steel beam decreased gradually. However, the internal force of the lower flange of the steel beam in the positive bending moment zone still increased because of the stiffness reduction in the negative moment zone.
(3) The internal force change in the composite girder was characterized by three stages. The stress changes during the first 10 days were significant, the stress change rate decreased continuously during days 10–90, and the stress changed slowly and smoothly after 90 days. The bending moment change in the stud along the longitudinal direction was characterized by a sinusoidal distribution. The stud bending moment was the largest in Section S3. The stud bending moment near the 3rd pier section increased gradually as a result of concrete creep, and the stud bending moments in the other sections decreased gradually.

## Figures and Tables

**Table 1:** Maximum temperature difference in the girder during each day.

		Temperature of Each Measuring Point (°C)											
Section	Day	SC1	SC2	SC3	SC4	SC5	SC6	SC7	SC8	SC9	SC10	SI1	SI2
S4	1	4.90	5.10	4.60	5.00	5.40	4.70	5.00	4.70	4.50	4.10	2.90	2.60
	2	11.40	12.60	11.60	12.40	11.50	11.90	11.60	12.20	10.20	8.30	8.20	7.40
	3	11.40	11.20	11.00	11.40	11.30	11.50	11.7	11.70	9.20	8.50	8.10	7.20
	4	9.90	11.60	10.90	11.50	11.40	11.50	12.30	9.90	10.80	9.00	9.20	8.90
	5	7.60	8.30	8.50	8.10	8.50	8.30	8.30	7.50	7.70	6.50	4.30	3.90
	6	7.60	8.40	8.10	8.40	8.30	8.60	8.40	7.40	7.60	6.30	4.40	4.00
	7	8.50	9.90	9.20	9.80	9.70	9.60	9.90	8.30	9.40	8.10	5.50	5.40
S6	1	4.90	5.10	6.60	--	6.70	6.20	6.50	5.50	5.00	5.60	4.80	4.60
	2	13.00	14.20	14.20	--	14.80	14.70	14.90	13.50	10.10	10.10	9.00	8.50
	3	10.70	12.40	12.40	--	12.60	13.70	14.20	13.40	13.70	12.90	10.00	9.40
	4	12.20	13.40	13.40	--	14.10	14.00	13.70	11.10	12.20	11.70	10.60	10.20
	5	8.70	9.50	9.50	--	9.60	9.90	9.30	8.80	8.30	7.50	6.50	6.10
	6	8.90	9.60	9.60	--	9.60	9.80	9.40	8.80	8.30	7.50	6.50	6.10
	7	9.90	11.40	10.70	--	11.50	11.70	11.60	10.60	10.40	10.80	8.90	8.70
Average		9.26	10.19	10.02	9.51	10.36	10.44	10.39	9.53	9.10	8.35	7.06	6.64

**Table 2:** Parameters of the creep degree function.

Specimen	*A*_0_	*A*_1_	*m*
Ordinary concrete	29.713	−15.847	29.491
Steel fiber concrete	29.025	−12.717	28.149

**Table 3:** Dates of concrete pouring.

Casting Segment	Casting Area	Date
I	Positive bending moment zone of the fourth span	9 a.m., 23 April 2018
II	Positive bending moment zone of the third span	9 a.m., 24 April 2018
III	Positive bending moment zone of the second span	2 p.m., 24 April 2018
IV	Positive bending moment zone of the first span	9 a.m., 25 April 2018
V	Negative moment zones of the third and fourth spans	2 p.m., 25 April 2018
VI	Negative moment zones of the second and third spans	9 a.m., 27 April 2018
VII	Negative moment zones of the second and first spans	12 a.m., April 27, 2018

**Table 4:** Comparison between the calculated and measured girder strains in Section S4.

Casting Segment	Calculated Strain (με)		Measured Strain (με)		Error Percentage (%)	
	Bottom Plate	Top Plate	Bottom Plate	Top Plate	Bottom Plate	Top Plate
I	−5.1	6.4	−3.5	4.4	45.71	45.45
II	16.9	−12.5	12.7	−14.6	33.07	−14.38
III	−62.7	57.5	−53.2	46.2	17.86	24.46
IV	−130.4	124.9	−110.4	100.0	18.12	24.90
V	−87.3	124.5	−101.4	93.2	−13.91	33.58
VI	−119.9	124.9	−130.1	109.5	−7.84	14.06
VII	−131.1	118.2	−161.3	130.5	−18.72	−9.43

**Table 5:** Comparison between the calculated and measured girder strains in Section S6.

Casting Segment	Calculated Strain (με)			Measured Strain (με)			Error Percentage (%)		
	Bottom Plate	Top Plate	Concrete Slab	Bottom Plate	Top Plate	Concrete Slab	Bottom Plate	Top Plate	Concrete Slab
I	−5.3	5.3	0.0	−8.4	6.5	0.0	36.90	18.46	--
II	11.4	−9.5	0.0	13.6	-9.5	0.0	16.18	0.00	--
III	−47.3	28.7	0.0	−58.5	31.8	0.0	19.15	9.75	--
IV	202.0	−50.9	−120.5	239.1	−73.9	−120.4	15.52	31.12	0.08
V	202.4	−51.0	−120.5	245.2	−62.0	−126.8	17.46	17.74	−4.97
VI	202.0	−50.8	−120.5	238.8	−61.8	−132.4	15.41	17.80	−8.99
VII	199.1	−45.9	−120.8	240.1	−75.9	−136.4	17.08	39.53	−11.44
